# Cabin1 domain-containing gene *picd-1* interacts with *pry-1/Axin* to regulate multiple processes in *Caenorhabditis elegans*

**DOI:** 10.1038/s41598-022-15873-5

**Published:** 2022-07-14

**Authors:** Avijit Mallick, Shane K. B. Taylor, Sakshi Mehta, Bhagwati P. Gupta

**Affiliations:** Department of Biology, McMaster University, 1280 Main Street West, Hamilton, ON L8S 4K1 Canada

**Keywords:** Gene expression, Gene regulation, Genetic interaction

## Abstract

The Axin family of scaffolding proteins control diverse processes, such as facilitating the interactions between cellular components and providing specificity to signaling pathways. While several Axin family members have been discovered in metazoans and shown to play crucial roles, their mechanism of action are not well understood. The *Caenorhabditis elegans* Axin homolog, *pry-1*, is a powerful tool for identifying interacting genes and downstream effectors that function in a conserved manner to regulate Axin-mediated signaling. Our lab and others have established *pry-1’*s essential role in developmental processes that affect the reproductive system, seam cells, and a posterior P lineage cell, P11.p. Additionally, *pry-1* is crucial for lipid metabolism, stress responses, and aging. In this study, we expanded on our previous work on *pry-1* by reporting a novel interacting gene named *picd-1* (***p****ry-1*-**i**nteracting and **C**abin1 **d**omain-containing). PICD-1 protein shares sequence conservation with CABIN1, a component of the HUCA complex. Our findings have revealed that PICD-1 is involved in several *pry-1*-mediated processes, including stress response and lifespan maintenance. *picd-1*’s expression overlapped with that of *pry-1* in multiple tissues throughout the lifespan. Furthermore, PRY-1 and PICD-1 inhibited CREB-regulated transcriptional coactivator homolog CRTC-1, which promotes longevity in a calcineurin-dependent manner. Overall, our study has demonstrated that *picd-1* is necessary for mediating *pry-1* function and provides the basis to investigate whether Cabin-1 domain-containing protein plays a similar role in Axin signaling in other systems.

## Introduction

Signaling pathways enable cells to communicate with each other and their environment. Because of their essential role in cells, these pathway components are tightly regulated via interaction with a host of cellular factors. The Axin family is a group of scaffolding proteins that bring together different proteins to facilitate their interactions and regulate their activity^1^. There are two Axin homologs in mammals: Axin1 and Axin2. Axin was initially discovered as a negative regulator of the WNT-mediated signaling cascade. However, subsequent studies revealed a much broader role of these proteins in other pathways, including JNK, AMPK, and TGFβ^1–4^. Axin regulates several processes, including organogenesis, anterior–posterior axis formation, neuronal development, and metabolic homeostasis. Further, loss of Axin function causes lethality, neuroectodermal defects, abnormal body axis patterning, and reduced adipogenesis^1^. However, the mechanism by which Axin regulates different biological processes and mediates specific interactions is not well understood.

In the nematode *C. elegans*, the Axin homolog PRY-1 controls processes such as embryogenesis, neuronal differentiation, vulval development, P11.p cell fate, and seam cell development^1,5–7^. The WNT-dependent function of PRY-1 in vulval cells involves its interactions with several other proteins to form a destruction complex that results in the phosphorylation and degradation of BAR-1 (β-Catenin)^5^. However, little is known about the factors that interact with PRY-1 in WNT-independent processes. A comprehensive understanding of *pry-1* function will require identification of its interacting proteins and downstream effectors. To this end, we previously performed a transcriptome profiling of *pry-1* that revealed novel interacting partners and genetic network of *pry-1* that regulate post-developmental events^6,8^. Specifically, we showed that PRY-1 is crucial for lipid metabolism, stress response, and lifespan maintenance, where it interacts with WNT-independent signaling pathway components^4,8–11^. These include SBP-1/SREBP and vitellogenin involved in fatty acid synthesis and lipid storage, AAK-2/AMPK in the muscle that non-autonomously activates DAF-16/FOXO in the intestine and delays aging^8,10^, and the components of the endoplasmic reticulum unfolded protein response (ER-UPR) pathway. Additionally, in a separate study, we reported genes regulated by *pry-1* during stress response and lifespan maintenance that include *cpz-1* (proteolysis), *cdk-1* (cell cycle), *rnr-1* (DNA replication), *his-7* (gene expression), and *ard-1* (mitochondrial oxidation/reduction)^7^.

This paper reports a novel downstream effector of *pry-1* signaling called *picd-1* that is critical for regulating multiple developmental and post-developmental processes. PICD-1 shares a domain with the mammalian calcineurin-binding protein 1 (CABIN1), a component of the histone H3.3 chaperone complex HUCA^12^. cabin1 negatively regulates calcineurin signaling, which in turn affects various cellular functions, including stress response and lifespan^13–16^. We show that PICD-1 negatively regulates CREB-regulated transcriptional coactivator (CRTC) homolog, CRTC-1, which promotes longevity mediated by calcineurin signaling^17^. Consistent with the *pry-1*’s role in promoting *picd-1* expression, *pry-1* mutants exhibit nuclear localization of CRTC-1, suggesting that PICD-1 is involved in PRY-1-mediated CRTC-1 regulation. These results demonstrate the critical role of PICD-1 in *C. elegans* and prompt future studies to investigate the involvement of Cabin1 and calcineurin signaling in Axin-mediated processes in eukaryotes.

## Results

### *picd-1* encodes a CABIN1 domain-containing protein

**Figure 1:**
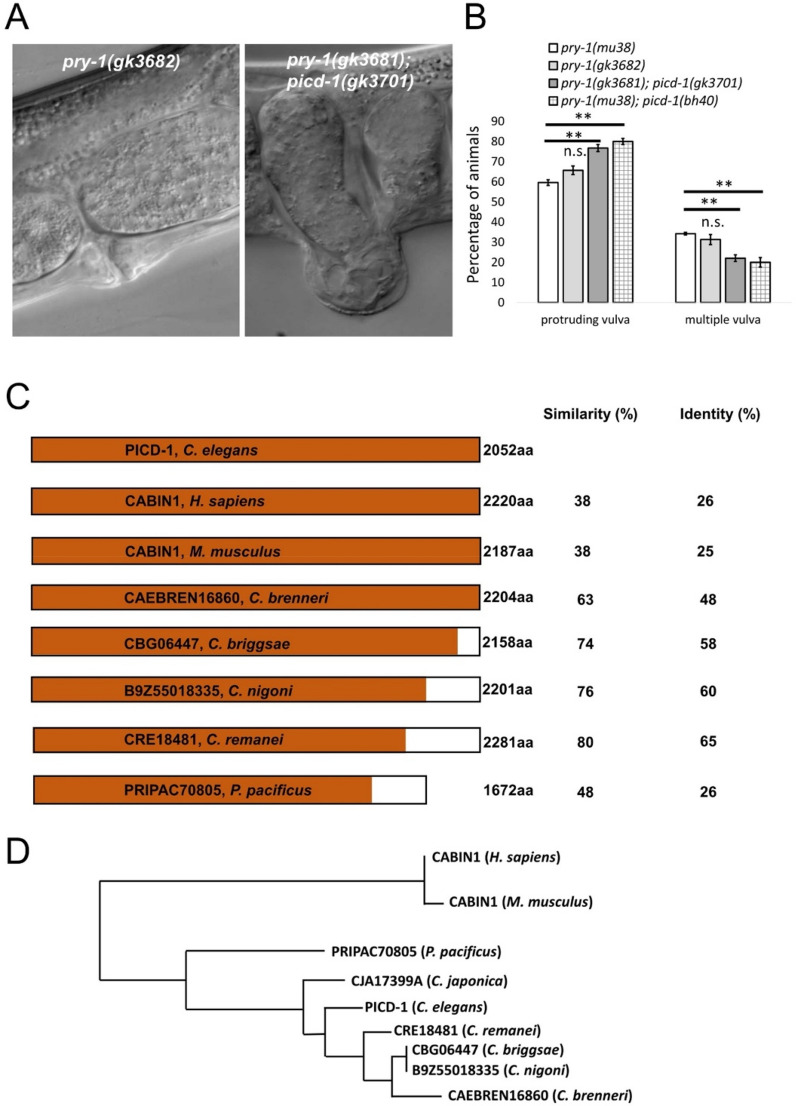
Phenotype of *picd-1* mutants and sequence similarity of PICD-1 with CABIN1 proteins. (**A**) Representative images of Pvl phenotype in *pry-1(gk3682)* and *pry-1(gk3681); picd-1(gk3701)* animals. (**B**) *picd-1* mutation enhances Pvl phenotype of *pry-1* mutants. Data represent a cumulative of two replicates (n = 99 for *pry-1(mu38),* 160 for *pry-1(mu38); picd-1(bh40)*, 118 for *pry-1(gk3682)* and 156 for *pry-1(gk3682); picd-1(gk3701)*) and error bars represent the standard deviation (Also see Table 1 and Fig. 3). Statistical analyses for panel (**B**) were done compared to *pry-1* mutants alone using one-way ANOVA with Dunnett’s post hoc test and significant differences are indicated by stars (*): ** (*p* < 0.01). n.s., not significant. (**C**) Sequence comparison of *C. elegans* PICD-1 with mammalian CABIN1 and nematode homologs. Orange colors indicate regions of the proteins where alignments were identified by NCBI BLAST along with similarity and identity scores. Non-colored regions are those without sequence similarity. (**D**) Sequence alignment dendrogram generated by LIRMM (http://www.phylogeny.fr/simple_phylogeny.cgi) using default parameters.

A CRISPR-based screening was carried out earlier to isolate alleles of *pry-1*^6^ (see “Methods”). In that screen we also discovered a mutation (*gk3701*) in an unrelated gene *F56E10.1* (WBGene00018975), now named *picd-1* (***p****ry-1* **i**nteracting and **C**abin1 **d**omain-containing, see “Methods”). The *pry-1(gk3681); picd-1(gk3701)* double mutant exhibited a significant increase in the protruding-vulva (Pvl) phenotype (77%, compared to 66% in *pry-1* mutants alone) and pronounced protrusions that frequently burst in the vulva (Table 1, Fig. 1A,B, Video S1). It should be noted that *gk3681* is identical to another *pry-1* mutation, *gk3682,* and both mutant strains were recovered in the same CRISPR screen (see “Methods” and Figure S1). Sequence analysis of *picd-1* identified orthologs in other nematode species (Fig. 1C), all of which contain a domain similar to the histone transcription regulator 3 (Hir3)/calcineurin-binding protein (CABIN1) family members (IPR033053, https://www.ebi.ac.uk/interpro/) (Fig. 1C,D). The alignments of PICD-1 with human CABIN1 (isoform a) showed 26% (729/2853) identity and 38% (1080/2853) similarity (EMBOSS stretcher pairwise alignment tool; https://www.ebi.ac.uk/Tools/psa/). Similar sequence conservation was observed in the case of mouse CABIN1 (Fig. 1C). Studies have shown that human CABIN1 is part of the histone H3.3 chaperone complex HUCA (HIRA/UBN1/CABIN1/ASF1a), involved in nucleosome assembly. Similarly, Gene Ontology (GO) analysis (http://www.wormbase.org) revealed that *picd-1* is associated with the biological process “DNA replication-independent nucleosome assembly” (GO:0006336) and the cellular component “nucleus” (GO:0005634). Thus, *picd-1* is likely to encode a nuclear protein that functions in chromatin assembly and regulation of gene expression. Furthermore, in silico analysis revealed that PICD-1 contains 49 amino acid residues predicted to bind DNA (http://biomine.cs.vcu.edu/servers/DRNApred/#References)^18^(Table S1).

**Table 1:** Analysis of VPC induction, P12.pa cell fate, Pvl, and Muv penetrance in different strains.

Genotype	VPC induction (% induced)							Over-induced VPCs and P12.pa			Pvl and Muv		
	P3p	P4p	P5p	P6p	P7p	P8p	N	% Over induced	% P12pa*	N	% Pvl	%Muv	N
N2	0	0	100	100	100	0	20	0	0	20	0	0	20
*pry-1(gk3682)*	27.3	4.5	100	100	86.4	4.5	22	36.4	72.7 (< 0.01)	22	65.7 (< 0.001)	31.3 (< 0.001)	40
*pry-1(mu38)*	18.2	0	100	100	81.8	9.1	22	27.2	81.8 (< 0.01)	22	59.6 (< 0.001)	34.2 (< 0.001)	50
*picd-1(gk3701)*	0	0	100	100	100	0	20	0	0 (n.s.)	20	5 (< 0.05)	0 (n.s.)	40
*picd-1(bh40)*	0	0	100	100	100	0	20	0	0 (n.s.)	20	16.7 (< 0.01)	0 (n.s.)	40
*pry-1(gk3681); picd-1(gk3701)*	21.7	8.7	100	100	87	13	23	39.1	74 (n.s.)	23	76.8 (< 0.01)	22 (< 0.01)	40
*pry-1(mu38)*	18.2	0	100	100	81.8	9.1	22	27.2	81.8	22	59.6	34.2	50
*pry-1(mu38); picd-1(bh40)*	12.5	12.5	100	100	92	12.5	24	25	100 (n.s.)	24	80 (< 0.01)	20 (< 0.01)	40

Each data consists of two independent replicates. Statistical analyses were done using one way-ANOVA. p values are indicated in brackets. n.s., not significant. While *pry-1* and *picd-1* mutants were compared to N2 control, *pry-1; picd-1* double mutants were compared to respective *pry-1* alleles. *Extra P12.pa cell in the place of P11.p. *N* number of animals examined.

### Mutations in *picd-1* do not affect vulval induction but cause morphogenetic defects

**Figure 2:**
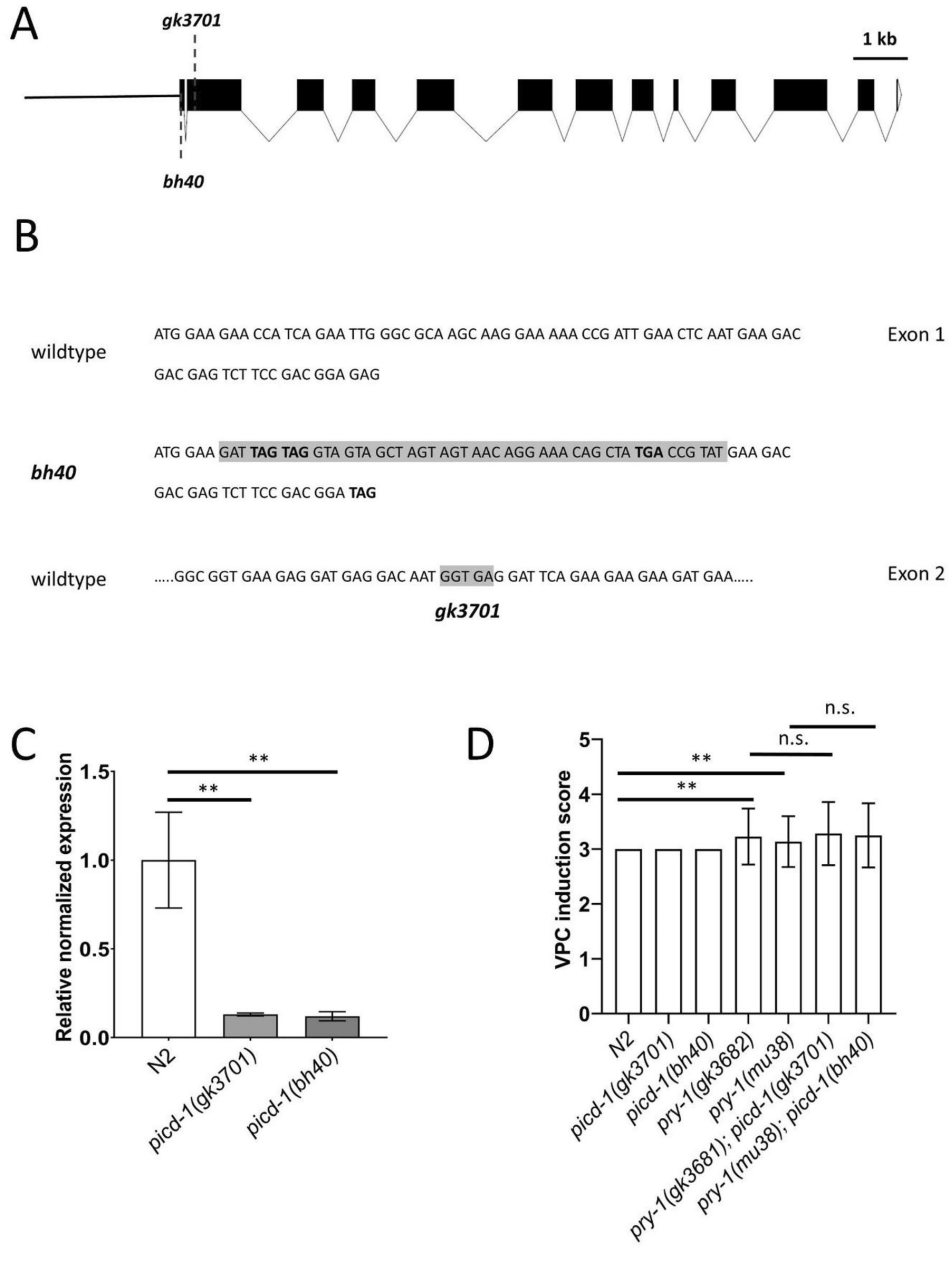
Analysis of *picd-1* alleles and their effect on *pry-1* mutant Pvl phenotype. (**A**) Schematic diagram of the *picd-1* open reading frame. The approximate locations of *bh40* and *gk3701* mutations have been indicated. (**B**) Sequences for *bh40* and *gk3701* mutations are shown in gray color. and stop codons are shown in bold (also see “Methods”). (**C**) Expression levels of *picd-1* in *pry-1(gk3682)* and *pry-1(mu38)* mutants at the L1 stage compared to wild-type. Data represent the means of two replicates and error bars represent the standard error of means. *p* values were calculated using Bio-Rad software (one-way ANOVA). (**D**) Bar graph showing VPC induction score in *picd-1* and *pry-1* mutants alone, and *pry-1; picd-1* double mutants compared to N2. Data represent the means of two replicates. The error bars show the standard deviation (n = 22). Statistical analyses were done using one-way ANOVA with Dunnett’s post hoc test. In panels (**C,D**), significant differences are indicated by stars (*): ** (*p* < 0.01). n.s., not significant.

**Figure 3:**
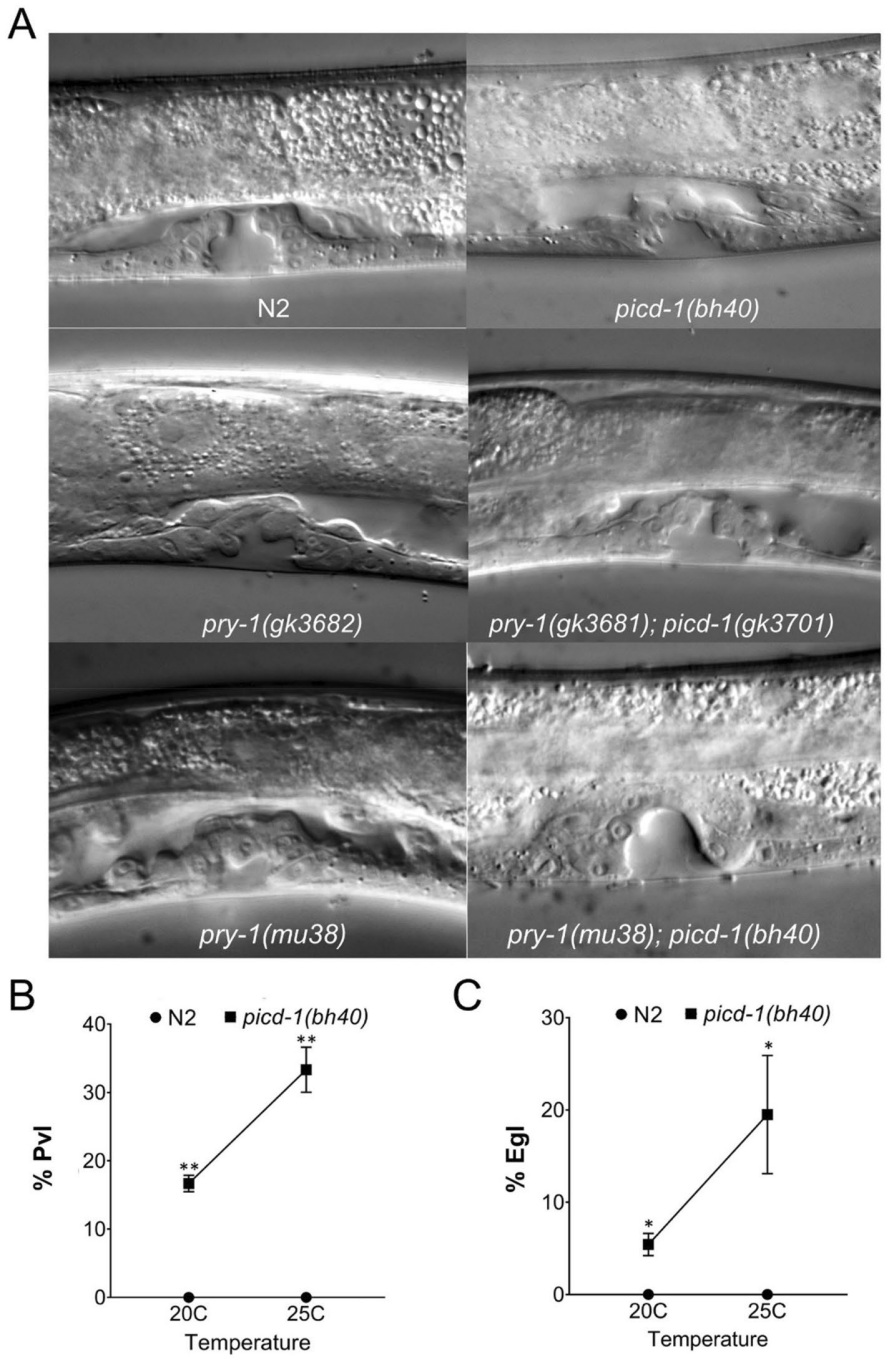
*picd-1* regulates vulval morphology. (**A**) Representative vulval images of wild-type, and *picd-1* and *pry-1* mutants at the mid-L4 stage. *picd-1(bh40)* animals exhibited abnormal vulva morphology (20.8 ± 5.8%, *p* < 0.01, n = 35) compared to N2 animals. Scale bar is 50 µm. (**B**) Line graph showing the percentage of control and *picd-1(bh40)* mutants showing Pvl phenotype at 20 °C and 25 °C compared to wild-type controls (Also see Table 1). (**C**) Line graph showing the percentage of control and *picd-1(bh40)* animals with Egl phenotype at 20 °C and 25 °C. (**B**, **C**) Data represent a cumulative of two replicates (n = 64 for *picd-1(bh40)* and 58 for N2) and error bars represent the standard deviation. Statistical analyses were done using an unpaired t-test and significant differences are indicated by stars (*): * (*p* < 0.05), ** (*p* < 0.01).

In addition to using the *gk3701* strain to examine mutant phenotypes, we generated a new allele, *bh40*, which has multiple in-frame stop codons in exon 1 (see “Methods” and Fig. 2A,B). qPCR analysis showed that *bh40* and *gk3701* greatly reduced *picd-1* transcript levels (Fig. 2C). Interestingly, while the Pvl phenotype of *pry-1(mu38)* was enhanced by both the alleles (Fig. 1A,B), neither had an obvious impact on the penetrance of the multivulva (Muv) phenotype in *pry-1(mu38)* animals. In fact, the double mutants showed a slightly less Muv phenotype compared to *pry-1(mu38)* alone (Table 1, Fig. 1B), which may be due to changes in morphogenetic processes as vulval precursor induction is not affected by any of the *picd-1* mutations (Fig. 2D, Table 1). Similar phenotypes were observed following *picd-1* RNAi (Figure S2). In agreement with this, *picd-1(bh40),* but not *picd-1(gk3701),* exhibited abnormal vulval invagination (Fig. 3A) and the vulval morphology phenotype of *pry-1(mu38); picd-1(bh40)* double mutant was more severe compared to *pry-1(mu38)* alone**.** Furthermore, adult *picd-1* mutants were weakly Pvl (Fig. 3B and Table 1).

Phenotypic analysis of both *picd-1* mutant strains did not reveal any obvious defects in movement, feeding, and other characteristics. However, careful examination showed that this gene is involved in the development of the egg-laying system. The *picd-1(bh40)* worms were weakly egg-laying defective (Egl) (Fig. 3C, Video S2), and their Egl and Pvl phenotypes were enhanced at 25 °C (Fig. 3B,C). No Egl phenotype was observed in the *picd-1(gk3701)* strain. While more experiments are needed to determine the molecular basis of phenotypic differences between the two alleles, the data collectively demonstrate that *picd-1* is required for the development of the reproductive system.

### *picd-1* is expressed in multiple tissues

**Figure 4:**
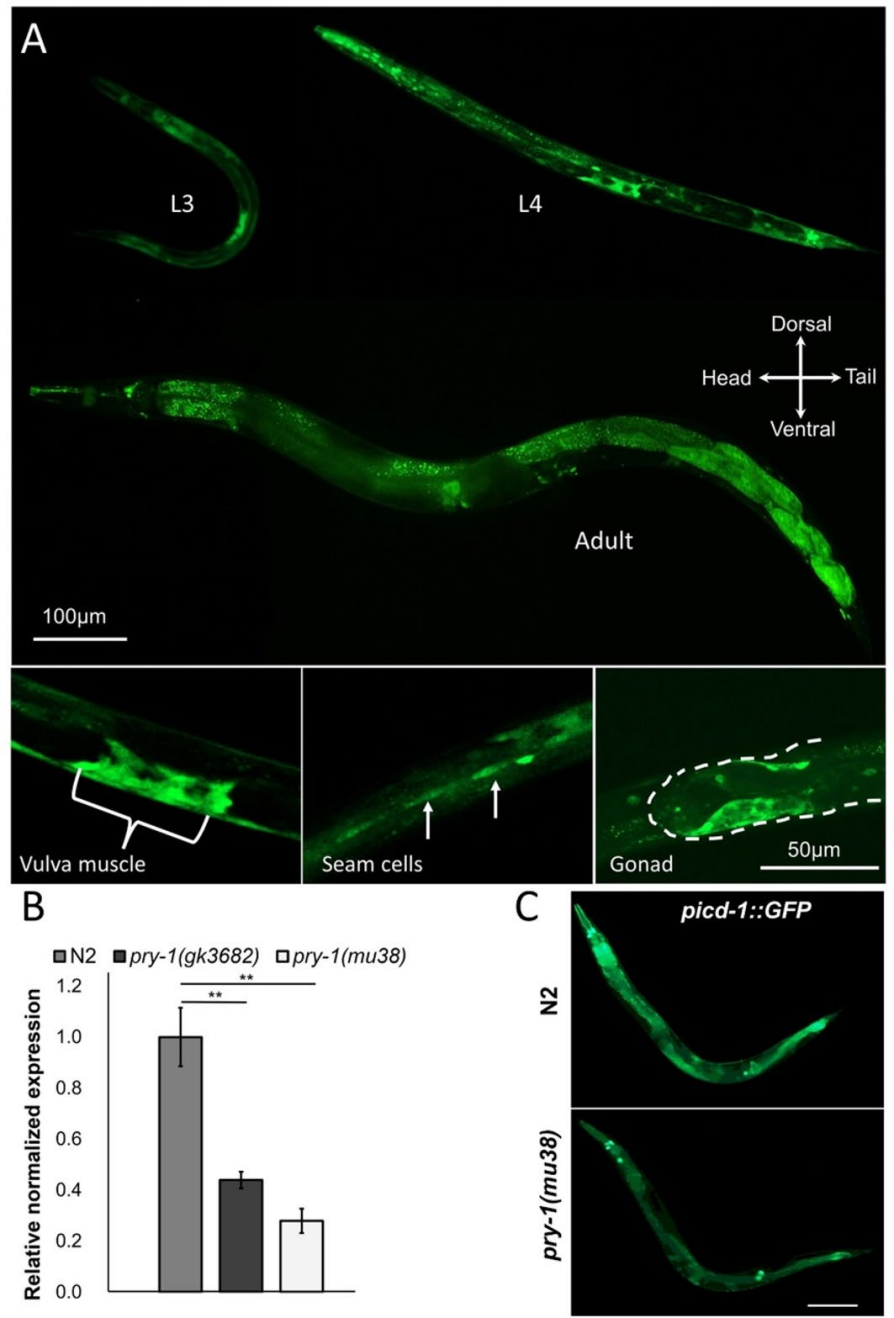
Expression analysis of *picd-1*. (**A**) Representative images of animals expressing *picd-1::GFP* in larvae and adults. Tissues that show fluorescence include pharynx, gonad, hypodermis, intestine, vulva, body wall muscles, and some in the tail region. Scale bars are shown. (**B**) Expression levels of *picd-1* in *pry-1* mutants. Data represent the means of two replicates and error bars represent the standard error of means. *p* values were calculated using Bio-Rad software (one-way ANOVA). In panel (**B**) significant differences are indicated by stars (*): ** (*p* < 0.01). (**C**) Representative images of N2 and *pry-1(mu38)* adults showing *picd-1::GFP* expression*.* Scale bar is 100 µm.

To gain further insights into the function of *picd-1*, we created a stable line carrying a *picd-1::GFP* transcriptional reporter. The analysis of transgenic animals revealed GFP fluorescence in developing tissues and organs, such as the pharynx, intestine, body wall muscles, hypodermis (seam cells), gonads, and vulva (Fig. 4A). This expression pattern resembled that of *pry-1*, which was recently described by our group^10^. As *picd-1::GFP* animals entered adulthood, fluorescence was localized to the intestine and certain head neurons (Fig. 4A), which persisted throughout the life of the animals. A broad range of *picd-1* expression was also supported by previously published RNA-sequencing and microarray studies^19,20^. Overall, our expression analysis suggests that *picd-1* functions in multiple tissues and may play a role in *pry-1*-mediated developmental and post -developmental processes.

Since *picd-1::GFP* is expressed in similar tissues as *pry-1,* and *picd-1* mutation enhanced the *pry-1* Pvl phenotype, we examined whether *pry-1* affects *picd-1* expression. The results of qPCR and *picd-1::GFP* fluorescence showed that *picd-1* is downregulated in *pry-1* mutants (Fig. 4B,C). The GFP levels were generally reduced throughout the animal (Fig. 4C). These results suggest that *picd-1* expression is dependent on *pry-1*.

### *picd-1* mutations worsen the phenotypes of *pry-1* mutants

**Figure 5:**
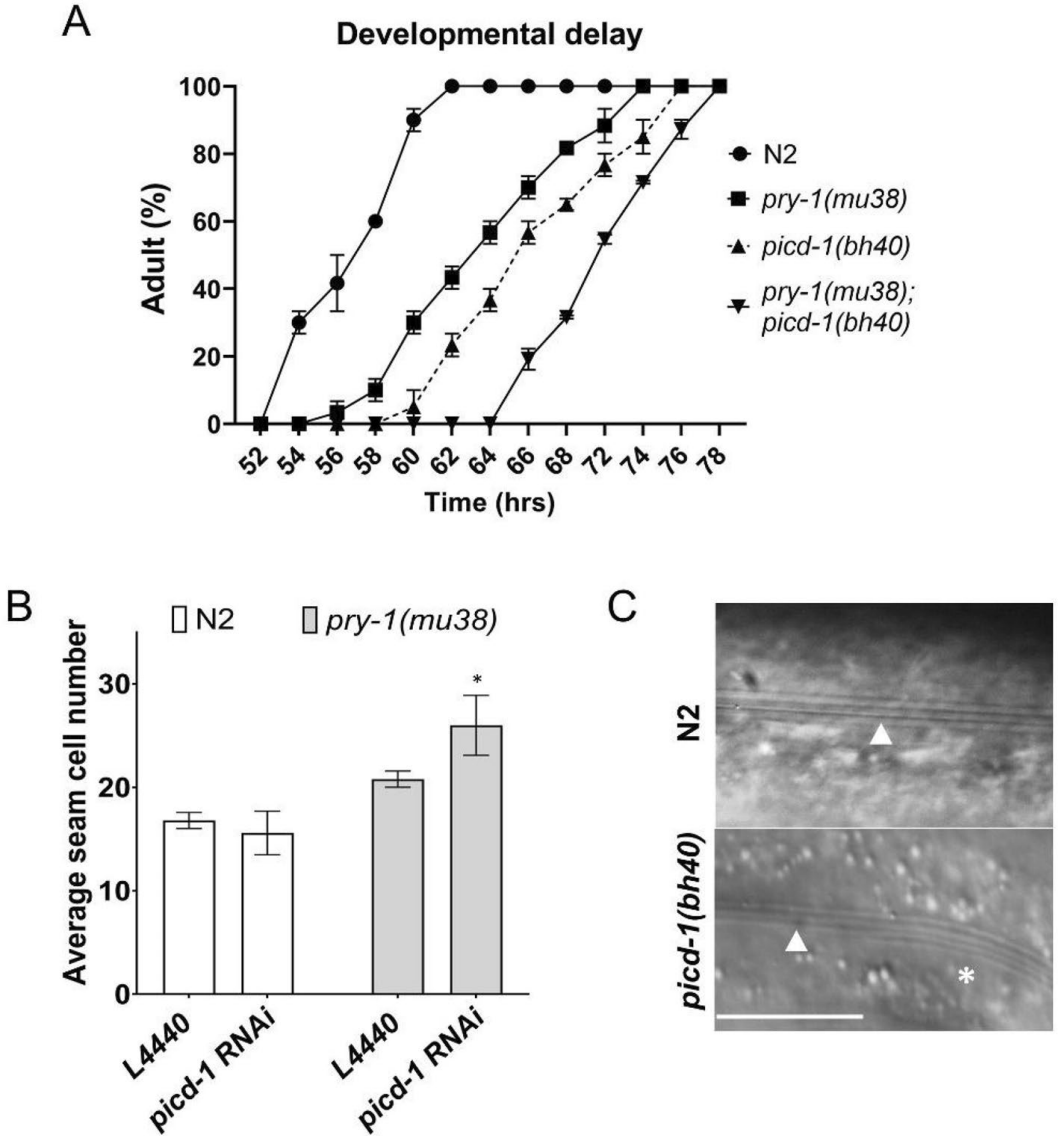
*picd-1* regulates developmental timing, seam cell division and alae formation. (**A**) *picd-1* mutants exacerbate the developmental delay of *pry-1* mutants. The data shows the average time taken by *picd-1(bh40), pry-1(mu38)* and *pry-1(mu38); picd-1(bh40)* double mutants to reach adulthood compared to wild-type animals. The number of animals for two replicates are: n = 60 for N2, *pry-1(mu38),* and *picd-1(bh40)*; and 95 for *pry-1(mu38); picd-1(bh40)*) The error bars represent the standard deviation. (**B**) Bar graph showing the average number of seam cells (two replicates, n = 30) in the wild-type and *pry-1(mu38)* animals following control (L4440) and *picd-1* RNAi. The error bars represent the standard deviation. (**C**) Representative images showing alae (white arrowheads) in wild-type N2 and *picd-1(bh40)* animals. Normal alae are marked with an arrowhead and an extra alae in the *picd-1* mutant is indicated by a star (*) (41.88 ± 0.22%, n = 43, *p* < 0.0001). Statistical analyses were done using multiple unpaired t-test and recorded in Table S2. In panel B, star (*) indicates *p* < 0.05. Scale bar in panel (**C**) is 25 µm.

Next, we investigated the involvement of *picd-1* in other *pry-1*-mediated developmental and post-developmental processes. *picd-1* mutants grew slowly and took longer to reach adulthood than the wild-type and *pry-1(mu38)* animals (Fig. 5A, Table S2). Furthermore, the growth defect in the *pry-1; picd-1* double mutant was significantly worse than in the single mutants (Fig. 5A, Table S2). Among other phenotypes, mutations in *picd-1* enhanced seam cell defects of *pry-1(mu38)* animals (Fig. 5B) that are caused by changes in asymmetric cell division at the L2 stage^6,21^. Moreover, both *picd-1* and *pry-1* mutants exhibited defects in alae, which are structures formed by differentiated seam cells^6^ (Fig. 5C). Interestingly, the P lineage defect in *pry-1* mutants that involves P11.p and P12.pa cells was enhanced in *pry-1(mu38); picd-1(bh40)* but not in *pry-1(gk3681); picd-1(gk3701)* animals (Table 1, also see “Methods”).

**Figure 6:**
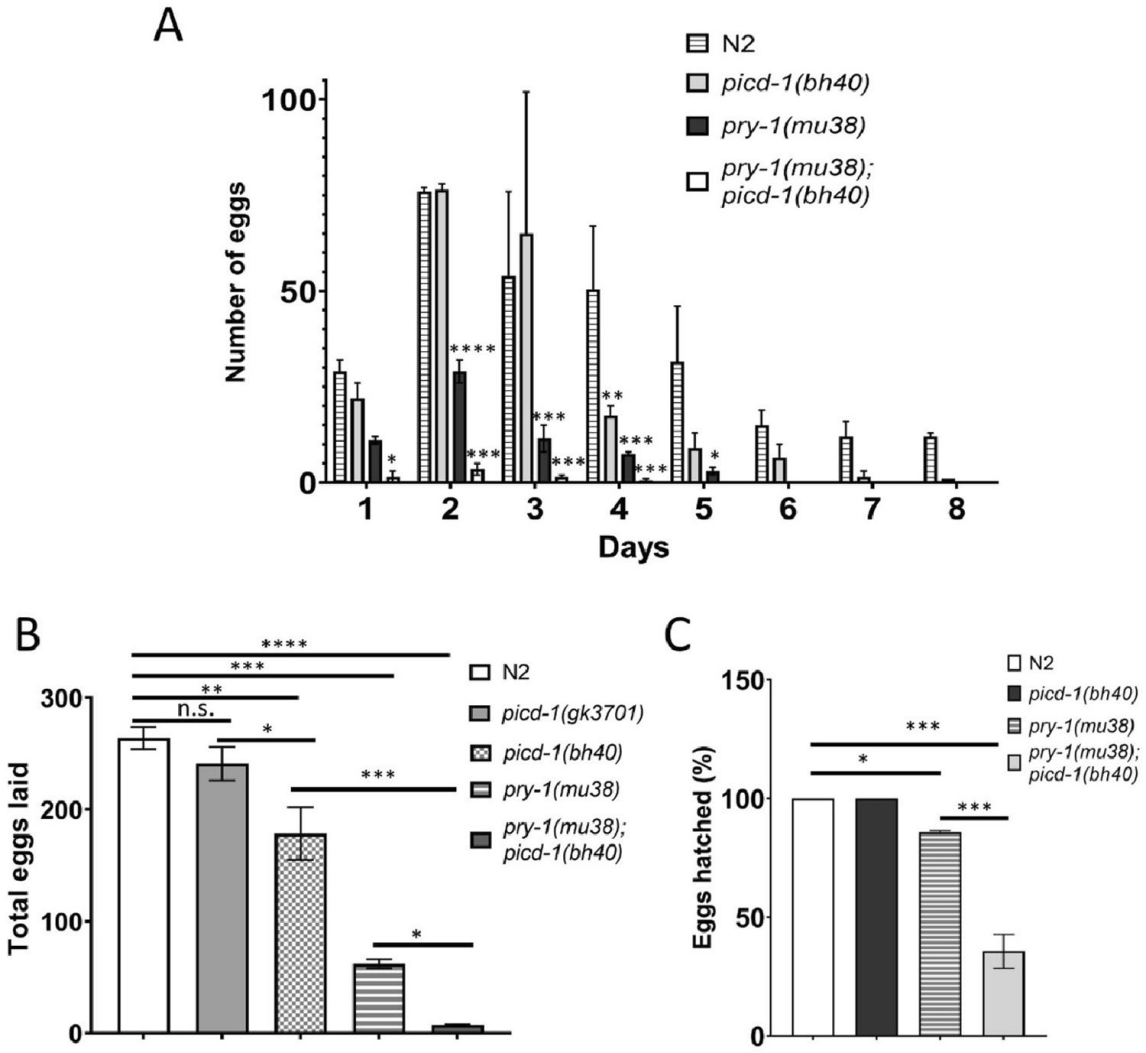
*picd-1* regulates brood size and embryonic viability. Bar graphs showing eggs laid on each day (**A**)**,** totals number of eggs (**B**), and percentage of the hatched eggs (**C**) by N2 and single and double mutant animals. Data represent a cumulative of two replicates (total n = 10 for each genotype shown in panels (**A**) and (**B**); and n = 500 for N2, 120 for *pry-1(mu38)* and 14 for *pry-1(mu38); picd-1(bh40)* double mutants shown in panel (**C**) and error bars represent the standard deviation. (**A**) Statistical analyses were done using two-way ANOVA with Tukey’s multiple comparison test and recorded in Table S2. (**B,C**) Statistical analyses were done using one-way ANOVA with Dunnett’s multiple comparison test and significant differences are indicated by stars (*): * (*p* < 0.05), ** (*p* < 0.01), *** (*p* < 0.001), and **** (p < 0.0001). n.s., not significant.

**Figure 7:**
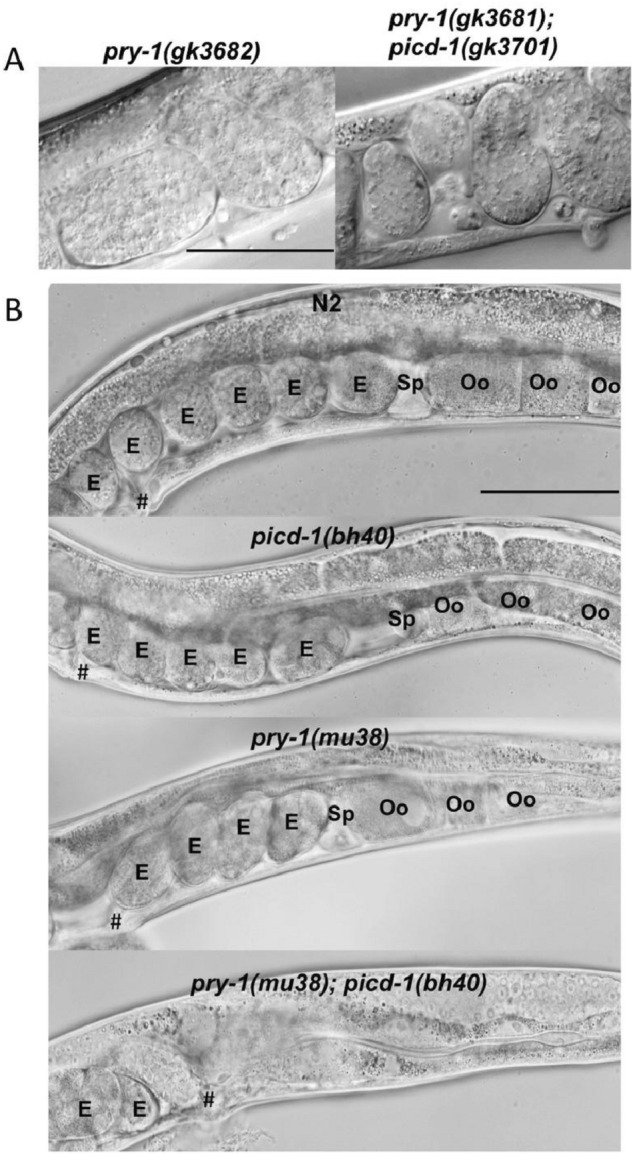
*picd-1* interacts with *pry-1* to regulate oocyte development. (**A**) *pry-1; picd-1* double mutants show abnormal oocytes. Scale bar is 25 µm. (**B**) Posterior gonad arms of wildtype, *picd-1(bh40), pry-1(mu38)* and *pry-1(mu38); picd-1(bh40)* adults. *pry-1(mu38); picd-1(bh40)* animals frequently lack oocytes (46 ± 6%, n = 45, *p* < 0.01) in the posterior gonad arm when compared to N2 and *pry-1(mu38)*. Statistical analysis was done using an unpaired t-test. The spermatheca (Sp), embryos (E), and oocytes (Oo) are marked. Vulva opening is marked (#). Anterior is on the left. Scale bar is 100 mm.

In addition, we observed several other developmental abnormalities in the *picd-1* mutant animals. The analysis of brood size revealed defects in *picd-1(bh40)* but not in *picd-1(gk3701)* animals (Fig. 6A,B). Although the *bh40* allele did not affect embryonic viability, it drastically reduced the brood count and enhanced the embryonic lethality of *pry-1* mutants (*p* < 0.001) (Fig. 6A–C). Further analysis revealed that *pry-1(gk3681); picd-1(gk3701)* and *pry-1(mu38)*; *picd-1(bh40)* double mutants had abnormal oocytes and gonads, respectively (Fig. 7A,B). More specifically, 46 ± 6% (n = 45, *p* < 0.01) of *pry-1(mu38)*; *picd-1(bh40)* animals lacked oocytes in the posterior gonad arm (Figs. 7B). No such phenotype was observed in either of the single mutants.

### *picd-1* mutants are sensitive to stress and exhibit a short lifespan

**Figure 8:**
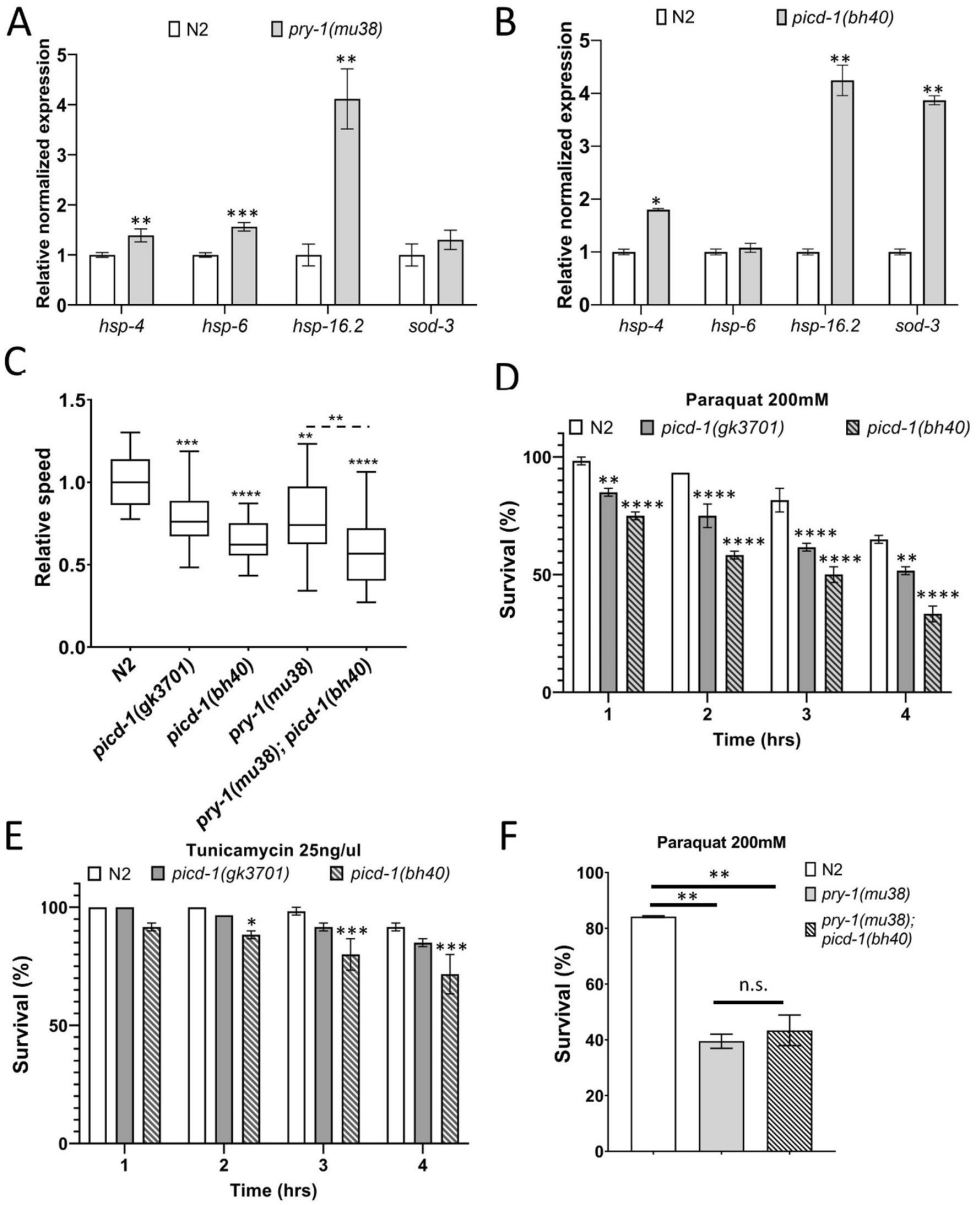
*picd-1* mutants are stress sensitive. (**A**, **B**) Expression levels of *hsp-4, hsp-6, hsp-16.2*, and *sod-3* in *picd-1(bh40)* and *pry-1(mu38)* young adults. Data represent the means of three replicates and error bars represent the standard error of means. *p* values were calculated using Bio-Rad software (t-test). (**C**) Box and whisker plots represent normalized electrotaxis speeds of *picd-1(gk3701)*, *picd-1(bh40)*, *pry-1(mu38)* and *pry-1(mu38); picd-1(bh40)* mutants (n = 20). Measurements show 25 to 75th percentiles of data, central horizontal lines represent medians, and vertical lines extend to 10th and 90th percentiles**. **(**D**) Bar graphs represent percentage survival following 200 mM paraquat exposure for 4 h (n = 90). (**E**) Bar graphs represent percentage survival of animals following 25 ng/µl tunicamycin exposure for 4 h. (**F**) Bar graphs represent percentage survival of animals following 200 mM paraquat exposure for 2 h (n = 120 for N2, 150 for *pry-1(mu38)* and 169 for *pry-1(mu38); picd-1(bh40)*). For panels (**C**–**F**), data are the cumulative of two replicates and error bars represent the standard deviation. (**C**, **F**) Statistical analyses were done using one-way ANOVA with Dunnett’s multiple comparison test. (**D**, **E**) Statistical analyses were done using two-way ANOVA with Tukey’s multiple comparison test. In all cases significant differences are indicated by stars (*): * (*p* < 0.05), ** (*p* < 0.01), *** (*p* < 0.001), **** (*p* < 0.0001). Refer to Table S2 for detailed statistical analyses.

We previously reported that *pry-1* plays a role in stress response maintenance^7,10^. The analysis of heat shock chaperones—*hsp-4* (ER-UPR), *hsp-6* (MT-UPR), and *hsp-16.2* (cytosolic heat shock response, HSR)—showed that all three were upregulated in *pry-1* mutant animals (Fig. 8A). Similar experiments in *picd-1* mutants showed increased expression of *hsp-4, hsp-16.2*, and the oxidative stress response gene *sod-3* (Fig. 8B). Consistent with these results, both *pry-1* and *picd-1* mutants showed electrotaxis defects (Fig. 8C and “Methods”), a phenotype observed in animals with abnormalities in stress sensitivity and UPR^22^.

To further elucidate the stress sensitivity of animals lacking *picd-1* function, we examined the survivability of the animals following chemical treatments. Both *gk3701* and *bh40* alleles were sensitive to paraquat and tunicamycin, although the effect was more pronounced following paraquat exposure (Fig. 8D,E). We also tested paraquat sensitivity of *pry-1(mu38); picd-1(bh40)* double mutants and found no difference compared to *pry-1(mu38)* alone (Fig. 8F), which could be explained by the significantly reduced expression of *picd-1* in *pry-1* mutants.

**Figure 9:**
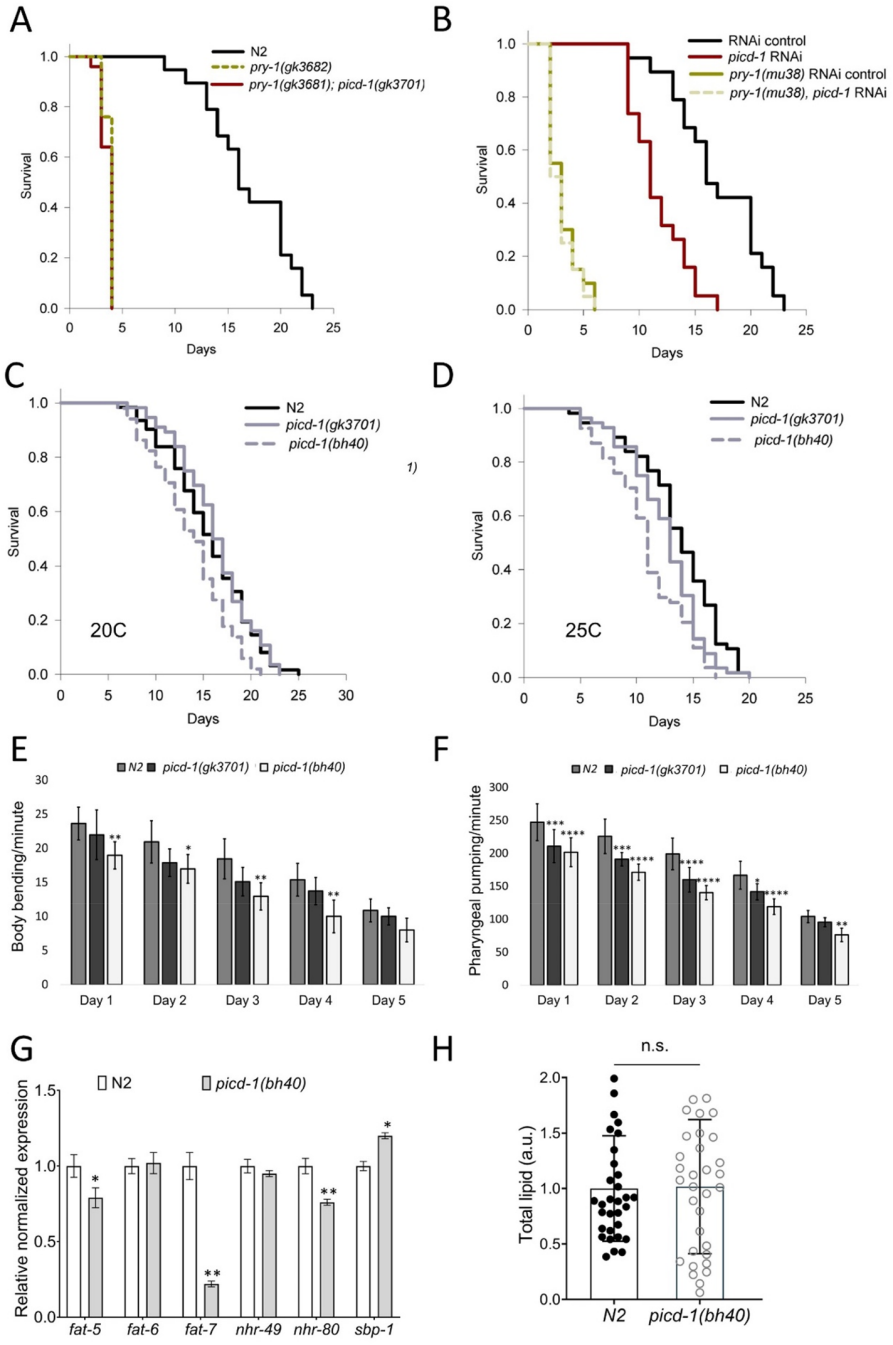
*picd-1* mutation reduces lifespan and causes age-associated deterioration. (**A**) *picd-1* mutation does not affect the lifespan of *pry-1* mutants. (**B**) *picd-1* RNAi reduces the lifespan of control animals but not that of *pry-1* mutants. (**C,D**) Lifespan of *picd-1(gk3701)* and *picd-1(bh40)* mutants at 20 °C and 25 °C. See “Materials and methods” section and Table 2 for lifespan data and statistical analyses. (**E,F**) Bar graphs showing the rates of body bending and pharyngeal pumping of *picd-1* mutants compared to wild-type over a period of 5 days. Data represent a cumulative of two replicates (n = 10 animals) and error bars represent the standard deviation. Statistical analyses were done using two-way ANOVA with Tukey’s multiple comparison test. See Table S2 for detailed statistical analyses. (**G**) Expression analysis of *fat-5, fat-6, fat-7, nhr-49*, *nhr-80* and *sbp-1* genes in the *picd-1(bh40)* mutants compared to wild-type. Data represent the means of two replicates and error bars represent the standard error of means. *p* values were calculated using Bio-Rad software (one-way ANOVA). (**H**) Quantification of total lipid using Oil Red O in the wild-type and *picd-1(bh40)* animals. Data represent a cumulative of two replicates (n > 30 animals) and error bars represent the standard deviation. Statistical analysis was done using an unpaired t-test. In all cases, significant differences are indicated by stars (*): * (*p* < 0.05), ** (*p* < 0.01), *** (*p* < 0.001), and **** (*p* < 0.0001).

As increased stress sensitivity can affect the lifespan of an animal, and *pry-1* mutants are short-lived, we analyzed whether *picd-1* plays a role in aging. Neither *picd-1*(*gk3701*) nor *picd-1*(RNAi) enhanced the lifespan defects of animals lacking *pry-1* function (Fig. 9A,B, Table 2). Considering that *pry-1* mutant animals have a significantly reduced expression of *picd-1* than the wild-type animals, it is conceivable that further reduction in *picd-1* cannot exacerbate the short-lived phenotype. Alternatively, it is plausible that *picd-1* is not involved in lifespan maintenance. To explore this further, we examined the lifespan of *picd-1* mutant and RNAi-treated animals in the absence of other mutations. Both *gk3701* and *bh40* alleles reduced the lifespan of the animals. *picd-1(bh40)* worms had a significantly reduced lifespan at both 20 °C and 25 °C, and *picd-1(gk3701)* exhibited a similar phenotype at 25 °C (Fig. 9C,D, Table 2). These results were also supported by the RNAi experiments. The analysis of age-associated biomarkers revealed a progressive age-associated decline in both body bending and pharyngeal pumping rates (Fig. 9E,F). Overall, the data suggest that while *picd-1* does not enhance the phenotype of *pry-1* mutants, the gene plays an essential role in maintaining the usual lifespan of animals.

**Table 2:** Lifespan analysis of animals.

Genotype	Treatment	Mean lifespan (days)	Median lifespan (days)	Maximum lifespan (days)	N	*p* value
N2	*–*	16.9 ± 0.9	16	23	56	–
*pry-1(gk3681)*	*–*	3.1 ± 0.2	3	5	86	< 0.0001
*pry-1(gk3682)*	*–*	3.7 ± 0.1	4	4	45	< 0.0001
*pry-1(gk3681); picd-1(gk3701)*	*–*	3.6 ± 0.1	4	4	50	< 0.0001
*picd-1(gk3701)*	*–*	15.3 ± 0.5	16	21	44	n.s
*picd-1(bh40)*	*–*	13.7 ± 0.5	14	21	51	< 0.001
N2	*Empty vector*	16.6 ± 0.9	16	21	75	–
	*picd-1 RNAi*	11.6 ± 0.6	18	22	58	< 0.001
	*crtc-1 RNAi*	20.4 ± 0.9	22	26	63	< 0.01
*pry-1(mu38)*	*empty vector*	3.1 ± 0.3	3	6	79	*–*
	*picd-1 RNAi*	2.9 ± 0.3	2	6	80	n.s
	*crtc-1 RNAi*	5.1 ± 0.4	6	8	48	< 0.001
*pry-1(gk3682)*	*empty vector*	4.1 ± 0.3	4	6	52	*–*
	*crtc-1 RNAi*	7.1 ± 0.7	7	10	64	< 0.001
N2	25 °C	13.4 ± 0.5	14	20	56	*–*
*picd-1(gk3701)*	25 °C	11.9 ± 0.5	12	16	48	< 0.001
*picd-1(bh40)*	25 °C	10.9 ± 0.4	11	17	54	< 0.001

Each lifespan assay was carried out in two or more batches (see Methods). Statistical analyses were done by comparing mutants with N2 control and RNAi treated animals with empty vector control. Comparisons of *pry-1(gk3681); picd-1(gk3701)* with *pry-1(gk3682)* and *picd-1(gk3701)* with *picd-1(bh40)* showed no significant differences. N, number of animals examined, n.s., not significant.

We have previously shown that in addition to affecting lifespan, *pry-1* regulates lipid metabolism^8,11^. This result prompted us to analyze whether *picd-1* affects lipid levels and the expression of genes involved in fatty acid synthesis. The analysis of ∆9 desaturases showed that while *fat-5* and *fat-7* were downregulated, *fat-6* was unaffected (Fig. 9G). Among the three transcription factors regulating the expression of ∆9 desaturases, *nhr-80* (NHR family) was downregulated, but *sbp-1* (SREBP1 homolog) was upregulated (Fig. 9G)^23^. We also quantified lipids by Oil Red O staining but saw no change in *picd-1* mutants (Fig. 9H), possibly due to functional redundancies within the *fat*^24,25^ and *nhr* family of genes^23^. Hence, we conclude that *picd-1* is necessary for the expression of a subset of lipid synthesis genes but is not crucial for regulating lipid levels.

### Loss of *picd-1* and *pry-1* promotes CRTC-1 nuclear localization

**Figure 10:**
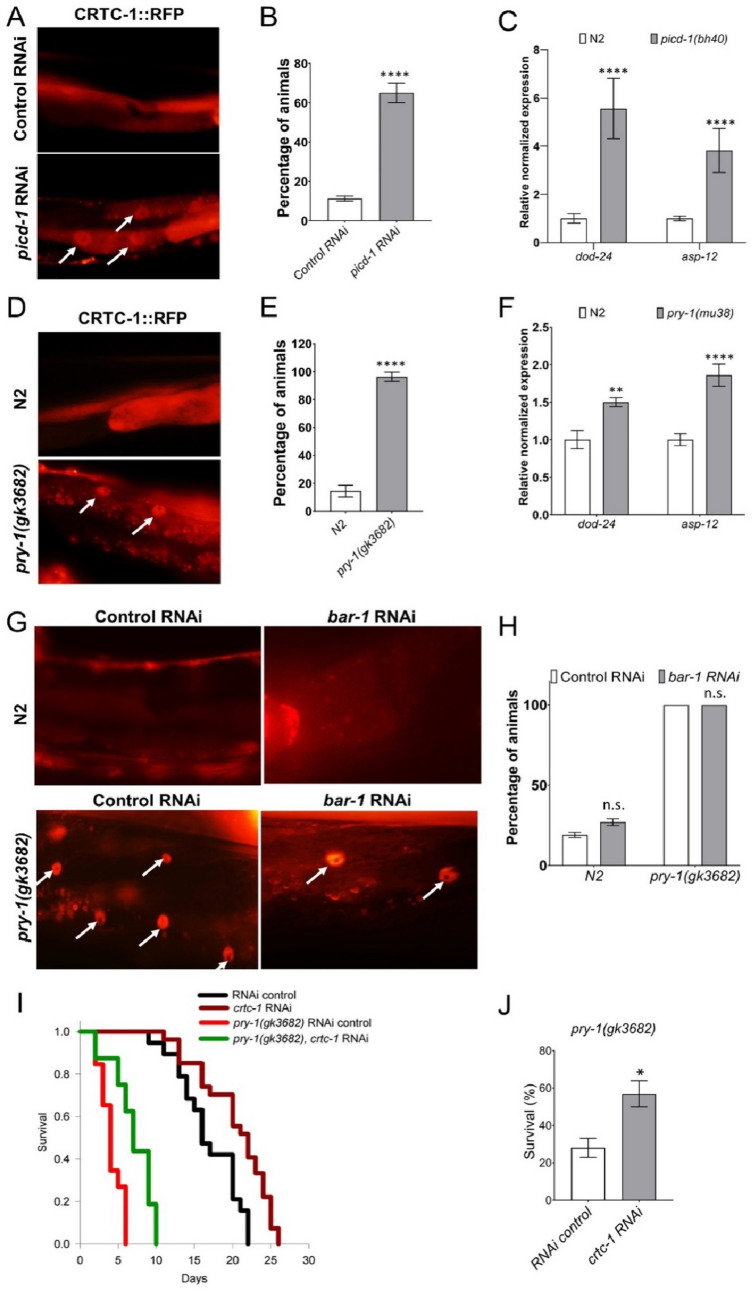
Reduced or loss of *picd-1* function affects CRTC-1 localization and CRTC-1 transcriptional response. (**A**) *picd-1,* but not L4440 control, RNAi causes nuclear accumulation of CRTC-1::RFP fluorescence. (**B**) Quantification of nuclear localization in (**A**)**. **(**C**) qPCR analysis of *dod-24* and *asp-12* in *picd-1(bh40)* animals shows increased expression. (**D–F**) Similar experiments performed in *pry-1* mutants. For panels (**C**) and (**F**), data represent the means of two replicates and error bars represent the standard error of means. (**G**) CRTC-1::RFP localization analysis in N2 and *pry-1(gk3682)* mutants following L4440 control RNAi, *bar-1* and *crtc-1* RNAi treatments. Nuclear fluorescence is absent in the case of *crtc-1* RNAi. (H) Quantification of nuclear localization in (**G**)**.** (**B,E**,**G**) Data represent the means of three replicates and error bars represent the standard deviation. At least n > 50 animals were examined in each assay. (**I**) Lifespan of wild type and *pry-1* mutant animals following L4440 control and *crtc-1* RNAi (also see Table 2). (**J**) Bar graphs represent percentage survival of animals following 100 mM paraquat exposure for 2 h. Data represent a cumulative of two replicates (n > 60 animals) and error bars represent the standard deviation. For panels (**B**,**E**,**G**,**J**), statistical analyses were done using an unpaired t-test. Data for panels (**C**) and (**F**) were analyzed using Bio-Rad software (t-test). In all cases, significant differences are indicated by stars (*): * (*p* < 0.05), ** (*p* < 0.01), and **** (*p* < 0.0001). n.s., not significant.

Research has shown that calcineurin (a calcium-activated phosphatase) signaling promotes nuclear localization of CRTC-1, leading to a reduction in the lifespan of *C. elegans*^17^. Given that human cabin1 negatively regulates calcineurin signaling^13,26^, we investigated whether *picd-1* could affect the subcellular localization of CRTC-1::RFP. The RNAi knockdown of *picd-1* caused CRTC-1 to be nuclear localized, consistent with the short lifespan of *picd-1* mutants (Figs. 9C,D, 10A,B). Moreover, CRTC-1 responsive genes, *dod-24* and *asp-12*^27^, were upregulated in the *picd-1(bh40)* mutants (Fig. 10C).

As *pry-1* is necessary for *picd-1* expression, we examined its effect on the CRTC-1::RFP and found that the fluorescence was nuclear-localized and *dod-24* and *asp-12* were upregulated in *pry-1* mutants (Fig. 10D–F). To further understand the mechanism of PRY-1-PICD-1-mediated CRTC-1 localization, we examined the involvement of the Wnt canonical pathway component BAR-1/β-catenin. The *bar-1* RNAi did not affect CRTC-1::RFP nuclear localization in *pry-1* mutants (Fig. 10G,H)**,** suggesting that PRY-1 may function in a WNT-independent manner to regulate CRTC-1. This conclusion agrees with our previous findings that *pry-1*-mediated lifespan maintenance does not depend on *bar-1*^10^. Next, we examined the involvement of CRTC-1 in PRY-1-mediated lifespan and stress response and found that *crtc-1* RNAi performed during the adult stage significantly rescued the short lifespan and stress sensitivity defects of *pry-1* mutants (Fig. 10I,J and Table 2). Hence, we propose that PRY-1 inhibits CRTC-1-dependent transcriptional response to delay age-associated processes.

Given that calcineurin and CRTC-1 mediated lifespan regulation involves the *C. elegans* CREB transcription factor homolog 1 (CRH-1), we wanted to know whether PRY-1 and CRH-1 regulate a common set of target genes. Consistent with our hypothesis, we found a significant overlap in differentially expressed genes between *pry-1* and *crh-1* mutant transcriptomes (406 common genes, R.F 2.5, *p* < 5.108e−78)^8,27^. Furthermore, the number of overlapping sets of genes regulated in an opposite manner between *pry-1* and *crh-1* mutants were also significant (Figure S3 and Table S3) and were enriched with GO biological processes such as response to stress (18), metabolic processes (18), and cellular processes (40) (FDR < 0.05). In conclusion, these data demonstrate a novel functional relationship between PRY-1 and CRTC-1 in *C. elegans*.

## Discussion

In this study, we identified a new gene, *picd-1*, in *C. elegans* that interacts with *pry-1* and regulates several larval and adult processes. *picd-1* is predicted to encode a nuclear protein containing a conserved Cabin1 domain, which belongs to the HUCA complex in humans^12^. The HUCA complex is implicated in diverse chromatin regulatory events, where it preferentially deposits a histone variant H3.3. This leads to transcriptional activation by nucleosome destabilization or transcriptional repression through heterochromatinization^28^. cabin1 is expressed in all human tissues and localized to the nucleoplasm and cytoplasm^29,30^. Studies in other systems have also uncovered homologous proteins of CABIN1. For example, the yeast *Saccharomyces cerevisiae* contains Hir1p and Hir2p (both HIRA orthologs) and Hir3, Hpc2, and Asf1p, orthologs of CABIN1, UBN1, and ASF1a, respectively^28^.

Our study provides the first genetic evidence of a Cabin1 domain-containing protein regulating biological processes in *C. elegans*. Other complex components in worms include HIRA-1 (HIRA homolog), ASFL-1, and UNC-85 (both ASF1a homologs)^31–33^. However, it remains to be seen if any of these proteins interact with PRY-1. Mutations in *picd-1* led to multiple defects such as Pvl, Egl, small brood size, developmental delay, stress sensitivity, and short lifespan. While there are differences in phenotypic severity between two *picd-1* alleles, it is important to emphasize that the loss of *picd-1* function enhanced various phenotypes of the *pry-1* mutant. For example, *pry-1; picd-1* double mutant showed a Pvl phenotype and exhibited P11.p cell fate changes. In addition, *picd-1* RNAi enhanced seam cell defects in the *pry-1* mutants. Interestingly, mutations in *picd-1* did not enhance vulval precursor cell induction or Muv phenotype in *pry-1* mutants. One possible model to explain genetic interactions between *pry-1* and *picd-1* is that *pry-1* functions via both *picd-1* dependent and independent pathways, and *picd-1* is regulated by multiple factors.

We analyzed the role of *picd-1* in *pry-1*-mediated non-developmental events, such as egg-laying, embryonic survivability, aging, stress response, and lipid metabolism. Loss of *picd-1* function worsened the embryonic lethality of *pry-1* mutants. Moreover, *pry-1; picd-1* double mutant had a very small brood size due to defects in the gonad arms. These findings suggest that *pry-1* and *picd-1* interact to regulate embryonic viability and fertility in animals. Similar phenotypes are observed in the mutants of other HIRA complex components. Knockdown of *hira-1* leads to embryonic lethality, *asfl-1* or *unc-85* single mutants have low brood size, and *asfl-1; unc-85* double mutant is sterile^32–34^. Together, these data show that *pry-1* and *picd-1* interact to regulate embryonic viability and fertility in animals. It remains to be seen whether PRY-1 and PICD-1 interact with other HIRA complex components to mediate their function.

Furthermore, we found that *picd-1* is required for normal stress response maintenance. *picd-1* mutants showed enhanced sensitivity to paraquat and tunicamycin. The mutant animals also exhibited increased levels of UPR markers. Both *picd-1* and *pry-1* mutants significantly increased *hsp-16.2,* and *hsp-4*, suggesting that these genes function together to regulate ER-UPR and HSR. However, more work is needed to determine whether these two genes uniquely affect MT-UPR and oxidative stress and their biological significance.

Mutants that show sensitivity to stress typically have a short lifespan^35–37^. Similar to *pry-1* mutants, *picd-1(bh40)* animals are short-lived and exhibit defects in age-related physiological markers. This result is consistent with the fact that both genes function together to regulate stress response and aging. However, there are functional differences between the two genes. For example, we found that lipid levels were greatly reduced in *pry-1* but not in *picd-1* mutants. The *nhr-80* and *fat-7* levels were reduced in *picd-1* mutant animals, consistent with the known role of *nhr-80* in regulating *fat-7* expression^24^. However, while *picd-1* is needed for the expression of *fat-5*, *fat-7* and *nhr-80*, a lack of its function does not compromise lipid levels in animals. These results are consistent with the above model of *picd-1* participating in a subset of *pry-1*-mediated processes. However, the extent to which the two genes interact and the precise nature of their interactions is unknown.

A possible mechanism of *picd-1* function in lifespan maintenance may involve calcineurin. AMPK and calcineurin modulation of CRTCs are conserved in mammals and *C. elegans*^17,38–41^. In *C. elegans*, AAK-2 and calcineurin regulate CRTC-1 post-translationally in an opposing manner, where activated AAK-2 causes nuclear exclusion of CRTC-1 and extends lifespan. Such a phenotype was also observed after deactivating calcineurin^17^. Our data showed that loss of *picd-1* function resulted in the nuclear localization of CRTC-1 and activated the CRTC-1 target genes. These findings, together with the fact that mammalian cabin1 inhibits calcineurin-mediated signaling^13,26,42^, allows us to hypothesize that PICD-1 regulates CRTC-1 via downregulation of calcineurin in *C. elegans.* According to this, loss of *picd-1*/cabin-1 is expected to increase calcineurin signaling, which may explain the shorter lifespan of *picd-1* mutants. Further experiments are needed to investigate the extent to which the regulatory relationship between cabin1 and calcineurin is conserved in *C. elegans*.

As *picd-1* is downregulated in *pry-1* mutants, and both genes are needed for the proper subcellular localization of CRTC-1 and its downstream targets, we speculate that PRY-1 and PICD-1 use CRTC-1 to regulate stress response and lifespan of animals. While there is no evidence for the interaction between mammalian Axin and CRTCs, studies have shown that CREB, which associates with CRTCs, is inhibited by Axin-GSK3β signaling^43,44^. Our work provides the first evidence of genetic interactions between *pry-1*, *picd-1*, and *crtc-1* in *C. elegans*, which has uncovered a novel crosstalk between Axin and calcineurin signaling. However, several questions remain unanswered. For instance, the components of *pry-1* signaling affecting CRTC-1 nuclear localization are unknown. In preliminary experiments, *tax-6* RNAi (calcineurin catalytic subunit) did not affect the *pry-1* phenotype; however, more experiments are needed to completely ascertain its requirements. Additionally, whether *picd-1* is regulated by *pry-1* in a WNT-dependent manner or it is co-regulated by *pry-1* and *aak-2*^4,10^ independently of WNT needs thorough investigation. Moreover, it is unknown whether other HUCA complex components interact with PICD-1 to mediate PRY-1’s role during stress response and lifespan, as well as whether PRY-1 and PICD-1 co-regulate a common set of targets during these processes. Further work is needed to investigate these questions and to gain a deeper understanding of the conserved mechanisms involved in Axin-CABIN1 signaling in eukaryotes.

## Materials and methods

### Worm strains

Cultures were maintained at 20 °C on standard nematode growth media (NGM) plates seeded with OP50 *E. coli* bacteria. The strains used in this study are listed below.

N2 (wild-type).

DY220 *pry-1(mu38).*

VC3710 *pry-1(gk3682).*

VC3709 *pry-1(gk3681); picd-1(gk3701).*

DY725 *pry-1(mu38); picd-1(bh40).*

DY678 *bhEx287[pGLC150(picd-1::GFP)* + *myo-3::wCherry].*

DY750 *bhEx301[pGLC150(picd-1::GFP)* + *pGLC 72(dat-1::YFP)].*

DY752 *pry-1(mu38); bhEx301[pGLC150(picd-1::GFP)* + *pGLC 72(dat-1::YFP)].*

DY698 and DY753 *picd-1(bh40).*

DY694 *picd-1(gk3701).*

DY747 *pry-1(gk3681).*

RG733 *wIs78[scm::GFP* + *ajm-1::GFP].*

AGD418 *uthIs205[crtc-1::CRTC-1::RFP::unc-54 3’ UTR* + *rol-6(su1006)].*

DY740 *pry-1(gk3682); uthIs205[crtc-1::CRTC-1::RFP* + *rol-6(su1006)].*

### Mutant alleles and transgenic strains

The *gk3701* mutation was discovered in a CRISPR screen that was carried out in Moerman lab to obtain alleles of *pry-1* and several other genes of interest^6,45,46^. Since the original *picd-1* mutant strain also contained *pry-1(gk3681)*, it was deconstructed to establish separate strains of *picd-1(gk3701)* (outcrossed 3x) and *pry-1(gk3681)* (outcrossed 2x). The presence of CRISPR mutations was confirmed by PCR and sequencing. Molecular changes in *pry-1(gk3682)* were described earlier by our group^6^. Both *gk3682* and *gk3681* carry a CRISPR GFP cassette^45^ We sequenced *gk3681* as part of this study and found that the allele is identical to *gk3682* (Table S4). In agreement with this, *pry-1(gk3681)* animals are phenotypically similar to *pry-1(gk3682)* (Figure S1 and Table 2).

The *picd-1(gk3701)* does not contain a CRISPR GFP cassette. Instead, it carries a 5 bp sequence (GGTGA) deletion in the second exon of the gene (flanking 25 nucleotides: GTGAAGAGGATGAGGACAATGGTGA and GGATTCAGAAGAAGAAGATGAAGAA) that causes multiple premature in-frame stop codons.

The other *picd-1* allele *(bh40)* was generated in a separate CRISPR screen using a published protocol^47^. Here, we replaced the 84 bp in the first exon by a synthetic sequence containing stop codons in different reading frames. See primers in Table S4. *bh40* animals were outcrossed 2 × and examined for various phenotypes. Another 2 × outcross of the strain was subsequently carried out (total 4x), which showed similar defects (Table S5).

To generate the *picd-1p::GFP* transgenic animals (DY678), pGLC150 was injected in N2 background along with *myo-3::wCherry* or *dat-1::YFP* marker. pGLC150 was constructed by cloning a 3,885 bp PCR-amplified fragment (using the primers GL1372 and GL1373), spanning the promoter region and a portion of the first exon of the *picd-1* gene, into the vector pPD95.81 using the restriction sites SalI and KpnI.

### Molecular biology

RNA was extracted from synchronized L3 and day-1 adult animals. Protocols for RNA extraction, cDNA synthesis and qPCR were described earlier^7^. Briefly, total RNA was extracted using Trizol (Thermo Fisher, USA). The RNA was used to prepare cDNA and, subsequently, perform qPCR using the SensiFast cDNA synthesis kit (Bioline, USA), and SYBR green mix (Bio-Rad, Canada), respectively. Primers are listed in Table S4.

### RNAi

RNAi mediated gene silencing was performed using a protocol previously published by our laboratory^48^. Plates were seeded with *E. coli* HT115 expressing either dsRNA specific to candidate genes or empty vector (L4440). Gravid hermaphrodites were treated with a mild bleach solution (3:2 ratio of commercial bleach and 4 N NaOH) and eggs were plated. Animals were subjected to RNAi treatment from egg stage unless otherwise stated. Phenotypes were examined at the young adult stage.

### Fluorescent and DIC microscopy

Animals were paralyzed in 10 mM Sodium Azide and mounted on glass slides containing 2% agar pads and covered with glass coverslips. Images were captured using a Zeiss Apotome microscope and Zeiss ZEN software. For acquiring live videos of gonad, animals were suspended in M9 without Sodium Azide. Videos were captured by a high-speed camera fitted on a Leica MZ-FLIII fluorescent stereomicroscope^49^.

Seam cells were identified using *scm::GFP* and *ajm-1::GFP* markers, The nuclei of these cells were counted, and adult lateral alae were scored using Nomarski differential interference contrast and epifluorescence optics. Images were acquired using a Hamamatsu Camera mounted on a Nikon Eclipse 80i upright Nomarski fluorescence microscope and NIS Element software (Nikon, USA).

### Vulval induction and P cells

Vulval induction and P11.p and P12.p phenotypes were examined in mounted L4 stage animals using a Nomarski microscope. VPCs were considered induced if they gave rise to progeny. Wild-type (N2) animals have three induced VPCs, one each for P5.p, P6.p, and P7.p. Mutants with more than three induced VPCs were termed as ‘over-induced’. Muv and Pvl phenotypes were scored in adults. Vulval morphogenesis is described previously^50^. At the L4 stage VPC progeny undergo a series of changes that involve invagination of vulva progeny and selective cell fusion to give rise to a characteristic morphology of the vulva. Defects in morphogenetic processes can lead to an abnormal shape of the vulva.

P11.p and P12.pa cells can be readily distinguished under a Nomarski microscope based on their nuclear size and morphology^51–53^. In *pry-1* mutants, two P12.pa-like cells are observed and P11.p is missing.

### Aging-related analysis

Lifespan experiments of RNAi-treated animals were carried out using a previously described protocol^10^. Synchronized eggs were allowed to grow on NGM OP50 seeded plates till the late L4 larval stage after which they were transferred to RNAi bacteria seeded plates to perform adult stage-specific knockdown. Cultures were screened daily for dead animals and surviving adults were transferred every other day till the progeny production ceased. Censoring was done for animals that had either escaped, burrowed into the medium, showed a bursting at the vulva, or had progeny hatching inside the uterus^54^.

Body bending per 1 min and pharyngeal pumping per 30 s were analyzed in young adults over a period of four days^7^. For this, individual hermaphrodites were placed on OP50 plates and examined under a dissecting microscope. Pharyngeal pumping was assessed by observing the number of pharyngeal contractions. For body bending, animals were stimulated by tapping once on the tail by a platinum wire. Each full sinusoidal motion was counted as one body bend. Only animals that moved actively within 1 min were included in the analysis.

### Stress assay and electrotaxis

Oxidative and endoplasmic reticulum mediated stress experiments were performed using 200 mM paraquat (unless specified) (Thermo Fisher Scientific, USA) and 25 ng/µl tunicamycin (Sigma-Aldrich, Canada) respectively. Animals were incubated for 1 h, 2 h, 3 h and 4 h, following a previous published protocol^6^. The final working concentrations were made in M9. At least 30 animals of each strain were tested in replicates. Means and standard deviations were determined from experiments performed in duplicate. Animals were considered dead if they did not respond to a platinum wire touch and showed no thrashing or swimming movement in M9. Moreover, dead animals usually had an uncurled and straight body shape in comparison to the normal sinusoidal shape of worms.

The electrotaxis assay protocol has been described previously^55^. Briefly, synchronized worms were introduced into a microfluidic channel and subjected to an electric field of 3 V/cm. Locomotory data was extracted from recorded videos using custom MATLAB-based worm tracking software. Electrotaxis speed data was plotted using box plots.

### Oil Red O staining

Neutral lipid staining was done on synchronized day-1 adults using Oil Red O dye (Thermo Fisher Scientific, USA) following a published protocol^11^. Quantifications were performed using ImageJ software as described earlier^56^.

### Statistical analyses

SigmaPlot 11 was used to plot lifespan data that provided detailed statistical analysis on survival curves. The curves were estimated using the Kaplan–Meier test and differences among groups were assessed using the log-rank test. qPCR data analysis was done using software (Maestro 3.1) that controls the Bio-Rad CFX qPCR machine. The software performs built-statistical analyses of t-test and one-way ANOVA. For all other assays, data from repeat experiments were pooled and analyzed together, and statistical analyses were done using GraphPad Prism 9. *p* values less than 0.05 were considered statistically significant. Data for all the Figure panels are reported in Table S5.

## Supplementary Information

Supplementary Information.

Supplementary Figure S1.

Supplementary Figure S2.

Supplementary Figure S3.

Supplementary Table S1.

Supplementary Table S2.

Supplementary Table S3.

Supplementary Table S4.

Supplementary Table S5.

Supplementary Video S1.

Supplementary Video S2.

## Data Availability

All data generated or analysed during this study are included in this published article (and its Supplementary Information files). Additional details are available from the corresponding author on reasonable request.
