# Supplementary material for: Cabin1 domain-containing gene picd-1 interacts with pry-1/Axin to regulate multiple processes in Caenorhabditis elegans
Source: Sci Rep. 2022 Jul 14;12:12029. doi: 10.1038/s41598-022-15873-5 (PMC9283418; doi:10.1038/s41598-022-15873-5)
Supplement: Supplementary file 1 — Supplementary Information. [file 41598_2022_15873_MOESM1_ESM.docx]

**Supplementary information**

**Cabin1 domain-containing gene *picd-1* interacts with *pry-1/Axin* to regulate multiple processes in *Caenorhabditis elegans***

Avijit Mallick, Shane K. B. Taylor, Sakshi Mehta, and Bhagwati P. Gupta

Department of Biology, McMaster University, 1280 Main Street West, Hamilton ON L8S 4K1, Canada.

**Supplementary Videos**

**Supplementary Video S1:** Enhanced protruding vulva of *pry-1(mu38); picd-1(bh40)* animal.

**Supplementary Video S2:** Egl defect of *picd-1(bh40)* mutants.

**Supplementary Tables (Microsoft Excel sheets)**

**Table S1:** PICD-1 amino acid residues predicted to bind DNA.

**Table S2:** Statistical analyses for Figures 5A, 6A, 8D, 8E, 9E and 9F.

**Table S3:** Overlapping sets of differentially expressed genes in *pry-1* and *crh-1* mutants that are regulated in an opposite manner.

**Table S4:** List of primers used in the study.

**Table S5:** Data for different batches of experiments.

**Supplementary Figures**

**Supplementary Figure S1.**

Both *pry-1* alleles, *gk3681* and *gk3682,* show similar phenotypes. **(A)** Bar graph showing the Muv penetrance in *pry-1(gk3681)* and *pry-1(gk3682)* mutants. Data represent a cumulative of three replicates (n = 60 for N2, 112 for *pry-1(gk3681)* and 118 for *pry-1(gk3682)* mutants) and error bars represent the standard deviation. Statistical analysis was done using one-way ANOVA and significant differences are indicated by stars (*): **** (*p* <0.0001). **(B)** Lifespan plot of *pry-1* mutants and N2. Also see Table 2 and Methods for statistical analysis.

**Supplementary Figure S2.**

*picd-1* RNAi enhances the Pvl phenotype of *pry-1(mu38)* animals. Bar graph showing the percentage of *pry-1* mutants showing Pvl and Muv phenotype following control and *picd-1* RNAi. Data represent a cumulative of two replicates (n > 30 animals) and error bars represent the standard deviation. Statistical analysis was done using an unpaired t-test and significant differences are indicated by stars (*): * (*p* <0.05), ** (*p* <0.01).

**Supplementary Figure S3.**

Common sets of differentially expressed genes in *pry-1* and *crh-1* mutant transcriptomes. Venn diagrams showing 406 DE genes that are shared between *pry-1* and *crh-1* mutants as well as overlapping genes with opposite expression trends.
