# Supplementary figures and images for: Cabin1 domain-containing gene picd-1 interacts with pry-1/Axin to regulate multiple processes in Caenorhabditis elegans
Source: Sci Rep. 2022 Jul 14;12:12029. doi: 10.1038/s41598-022-15873-5 (PMC9283418; doi:10.1038/s41598-022-15873-5)

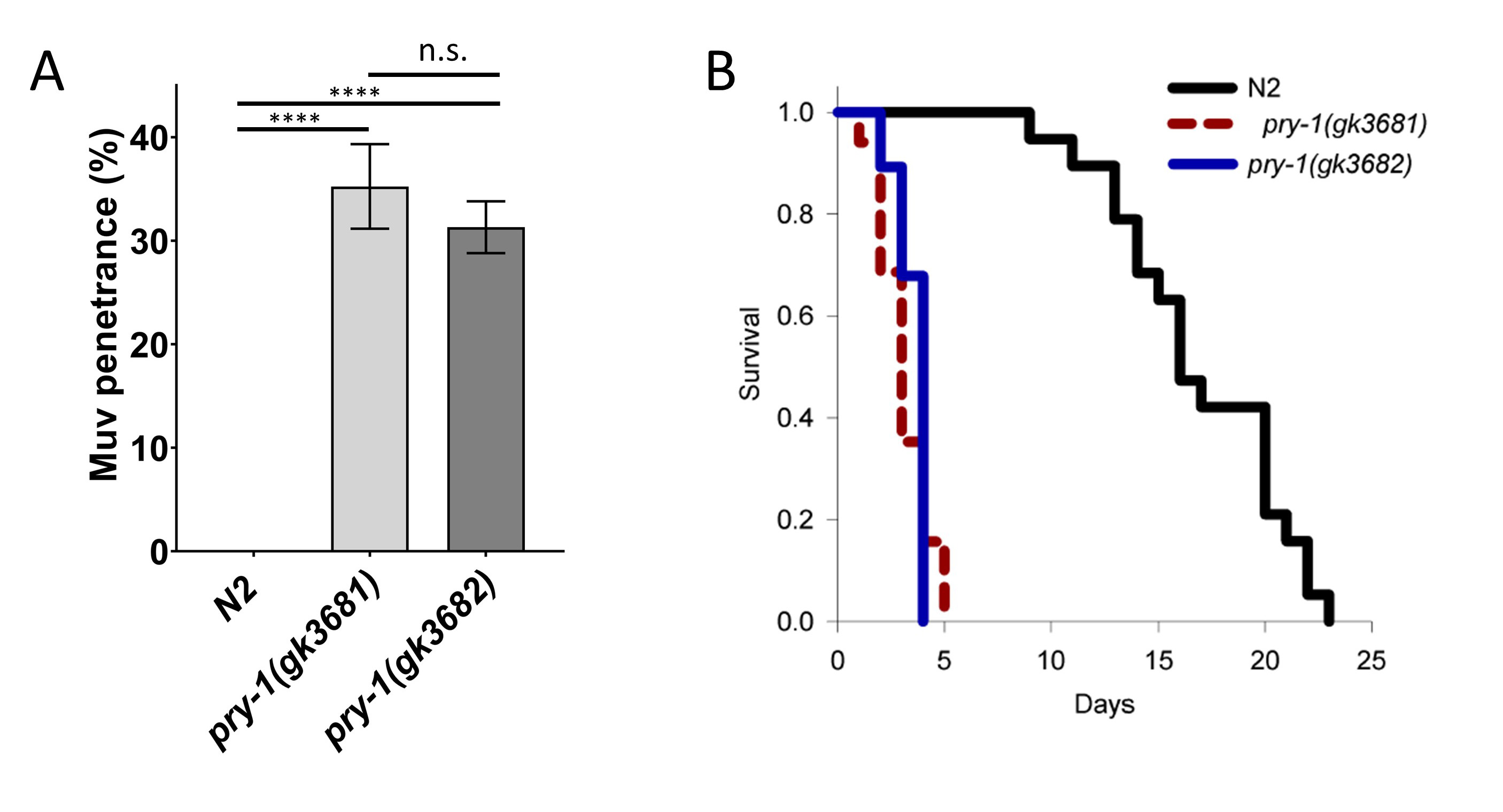

Supplement: Supplementary file 2 — Supplementary Figure S1. [file 41598_2022_15873_MOESM2_ESM.jpg]

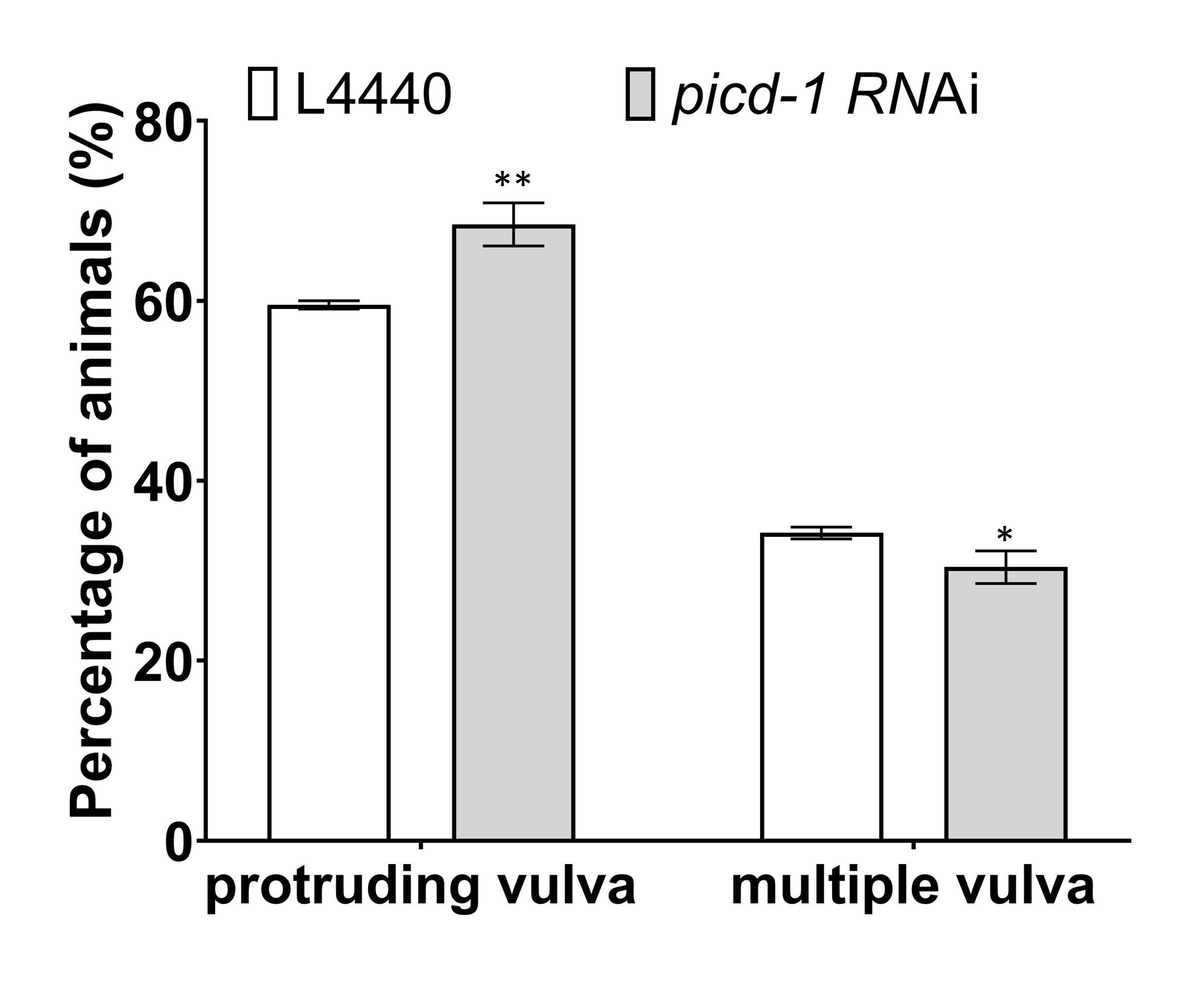

Supplement: Supplementary file 3 — Supplementary Figure S2. [file 41598_2022_15873_MOESM3_ESM.jpg]

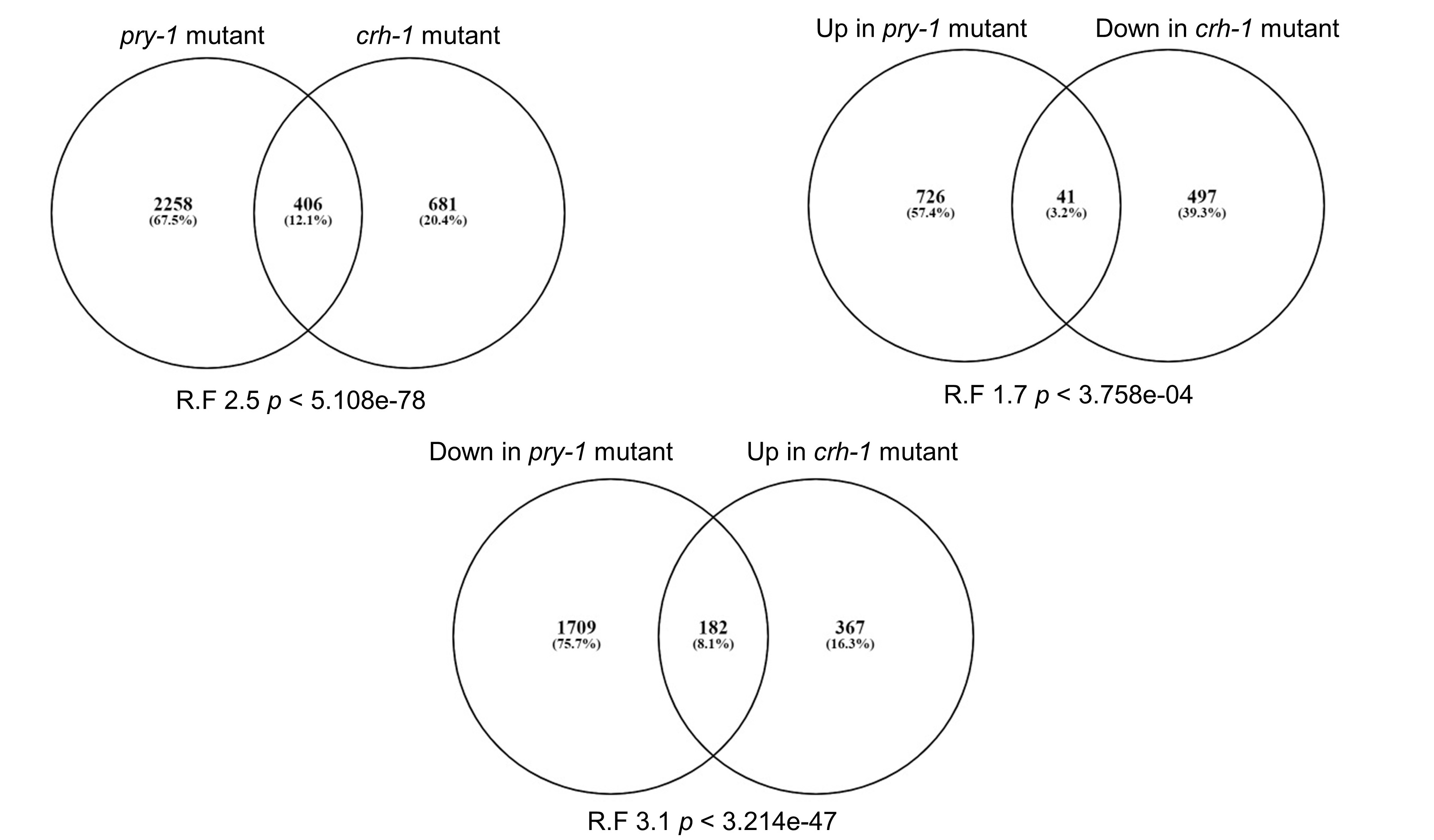

Supplement: Supplementary file 4 — Supplementary Figure S3. [file 41598_2022_15873_MOESM4_ESM.jpg]
